# A case report of a composite pheochromocytoma that occurred 13 years after adrenalectomy for pheochromocytoma

**DOI:** 10.3389/fonc.2026.1784221

**Published:** 2026-09-03

**Authors:** Min Min, Jie Zhang, Gui-xi Liu, Jing He

**Affiliations:** 1 Department of Pathology, The Third Hospital of Mianyang, Sichuan Mental Health Center, Mianyang, Sichuan, China; 2 Department of Radiology, The Third Hospital of Mianyang, Sichuan Mental Health Center, Mianyang, Sichuan, China; 3 Department of Urology Surgery, The Third Hospital of Mianyang, Sichuan Mental Health Center, Mianyang, Sichuan, China

**Keywords:** case report, composite pheochromocytoma, ganglioneuroma, pheochromocytoma, recurrence

## Abstract

**Purpose:**

To analyze the clinicopathological features, surgical management, and long-term prognosis of a composite pheochromocytoma (CP; pheochromocytoma combined with ganglioneuroma) arising 13 years after resection of a primary adrenal pheochromocytoma, with the aim of improving the clinical diagnosis and management of this rare neoplasm.

**Case presentation:**

A 61-year-old Han male had undergone left adrenal pheochromocytoma resection 13 years prior to the current presentation. An asymptomatic left adrenal nodule was incidentally detected during a routine physical examination, and the patient was subsequently admitted to the Department of Urology at The Third Hospital of Mianyang. He denied any history of other chronic systemic illnesses. Admission biochemistry revealed only a mild elevation of epinephrine, with the concentration remaining below twice the upper limit of normal. Abdominopelvic contrast-enhanced computed tomography (CT) identified a well-circumscribed hypodense nodule measuring 2.6 × 2.1 cm beneath the left adrenal gland, demonstrating marked nodular and peripheral ring enhancement; a separate hypodense nodule with a maximum diameter of 1.8 cm was also noted in the right adrenal fossa. Based on his disease history and imaging findings, recurrence of left adrenal pheochromocytoma was strongly suspected, and a synchronous right adrenal pheochromocytoma could not be excluded. The patient consented only to partial left adrenalectomy. Intraoperative exploration revealed adhesions among the descending colon, peritoneum, and spleen, as well as multiple solid masses (maximum size 2.0 × 2.0 cm) in the left adrenal region that were densely adherent to the pancreatic parenchyma. The resected specimens were submitted for pathological evaluation. Histopathological examination confirmed a composite pheochromocytoma with cystic change, multifocal coagulative necrosis, and adipose tissue invasion. The patient experienced an uneventful postoperative recovery. During the subsequent 11-month follow-up period, no recurrence or distant metastasis was detected, and the patient remained clinically stable.

**Conclusions:**

It remains challenging to definitively establish whether the left adrenal composite pheochromocytoma (CP) detected in this patient represents true tumor recurrence 13 years after the initial surgery. Archival pathological slides from the primary operation were unavailable for comparative histologic and clonal analysis. Moreover, the original lesion was diagnosed as a pure pheochromocytoma, whereas the current mass harbors an additional mature ganglioneuromatous component displaying distinct histological features. Given the lack of evaluable surgical margins from the initial partial adrenalectomy, the possibility of minimal residual disease cannot be excluded. Accordingly, two possibilities are proposed: late ipsilateral recurrence of the original chromaffin tumor, or a metachronous *de novo* primary CP arising independently in the left adrenal gland. In contrast to most previously documented CP cases, this patient exhibited isolated mild epinephrine elevation without catecholamine-related clinical manifestations, underscoring that regular physical screening and postoperative histopathology constitute the main diagnostic pillars. Surgical resection remains the first-line therapy. Regardless of its origin, long-term regular surveillance is mandatory, given the inherent metastatic potential of pheochromocytomas and paragangliomas (PPGLs).

## Introduction

Composite pheochromocytoma (CP) is a rare adrenal medullary neoplasm composed of conventional pheochromocytoma admixed with a distinct neuronal tumor component. The neuronal counterpart may include neuroblastoma (NB), ganglioneuroblastoma (GNB), ganglioneuroma (GN), or malignant peripheral nerve sheath tumor (MPNST), among others (1). The boundary between the two histological components is often poorly defined, with the pheochromocytoma element typically constituting the predominant portion of the tumor (2, 3). Among all composite subtypes, GN represents the most common neuronal component, accounting for approximately 75% of cases (4).

GN is a benign neurogenic tumor arising from sympathetic ganglia and is considered the fully differentiated, mature end-stage form of NB (5). It shows a female predominance and is most frequently diagnosed in patients under 20 years of age (6). CP, in contrast, accounts for 1%–9% of all pheochromocytomas (4), with a peak incidence between 40 and 50 years of age; no clear gender predilection has been identified, and the typical tumor diameter ranges from 4 to 6 cm. Affected individuals most commonly present with abdominal pain, a palpable abdominal mass, and a variety of cardiovascular or gastrointestinal symptoms (6). Adrenalectomy remains the gold-standard therapeutic intervention for these tumors.

Published clinical data on composite pheochromocytoma remain scarce, with fewer than 200 cumulative cases documented worldwide to date (7). Furthermore, it remains poorly defined whether the presence of an admixed neuroblastic component confers distinct therapeutic responses or prognostic risks compared with pure conventional pheochromocytoma.

This report describes an incidentally detected left adrenal composite pheochromocytoma admixed with GN. The patient presented without hypertension or any catecholamine-related clinical manifestations, despite a 13-year history of prior left adrenal pheochromocytoma resection. Preoperative imaging was highly suggestive of ipsilateral pheochromocytoma recurrence, although the possibility of a synchronous right adrenal pheochromocytoma could not be excluded. In accordance with the patient’s preference, surgical resection was limited to the left adrenal lesion.

Cases of ultra-late ipsilateral CP following prior pheochromocytoma surgery are rare. Although the majority of these neoplasms carry a favorable overall prognosis, they still confer a risk of local recurrence or metachronous *de novo* primary tumors within the adrenal glands, including the development of contralateral lesions. In the present case, a new left adrenal CP was identified in conjunction with a separate contralateral adrenal mass whose imaging characteristics were highly suggestive of pheochromocytoma. The left adrenal lesion was confirmed as CP by postoperative pathological examination. In light of the patient’s clinical history, this finding highlights the critical need to establish and implement standardized long-term surveillance protocols for patients with pheochromocytoma. Furthermore, routine standardized prognostic risk stratification should be adopted to optimize long-term survival outcomes in this patient population.

## Case presentation

A 61-year-old Han male presented to the Department of Urology at The Third Hospital of Mianyang after an incidental left adrenal mass was detected on routine physical examination one month prior to admission. He denied any associated clinical symptoms.

Thirteen years earlier, the patient had undergone open cortical-sparing partial left adrenalectomy at an outside hospital, with postoperative pathological examination confirming the diagnosis of pheochromocytoma. Given the prolonged interval since the initial surgery, the original medical records were no longer available, and baseline imaging data—including the maximum diameter of the primary tumor—could not be retrieved. The patient did not adhere to regular postoperative surveillance and sought no further medical attention until presenting for a routine physical examination at our institution.

The patient had no prior history of hypertension or other chronic illnesses, and there was no family history of hereditary neoplastic or endocrine disorders. Specialized physical examination revealed no percussion tenderness over either renal fossa, no tenderness along the bilateral ureteral tracts, and the absence of suprapubic bladder percussion tenderness.

**Figure 1:**
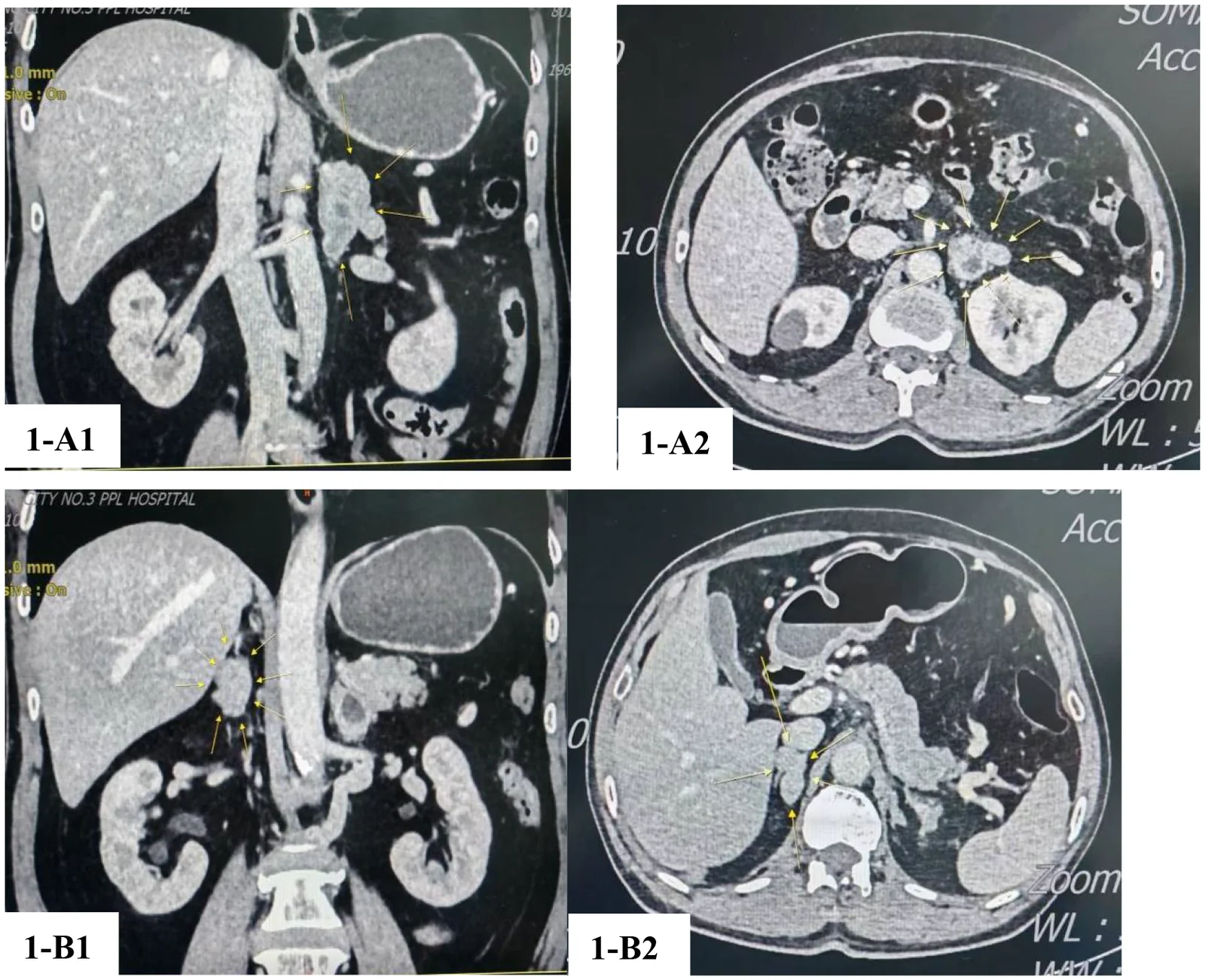
Contrast-enhanced abdominopelvic CT images. [**(A1)**, coronal plane; **(A2)**, axial plane] A well-circumscribed slightly hypoattenuating nodule is identified inferior to the left adrenal gland and medial to the mid-left kidney, demonstrating prominent nodular and peripheral ring enhancement (yellow arrow). [**(B1)**, coronal plane; **(B2)**, axial plane] A separate slightly hypoattenuating nodule is visualized within the right adrenal fossa (yellow arrow).*CT* computed tomography.

Morning hormone assays, performed on samples collected between 7:00 and 10:00 a.m., revealed an adrenocorticotropic hormone (ACTH) concentration of 39.88 pg/mL and a cortisol level of 10.98 μg/dL; both values were within the institutional normal reference ranges. Three serial plasma catecholamine measurements were conducted using high-performance liquid chromatography coupled with tandem mass spectrometry, with blood specimens obtained while the patient remained in the supine position. The laboratory findings were as follows: dopamine (DA) < 65.2 pmol/L, epinephrine (E) = 1081.9 pmol/L, and norepinephrine (NE) = 1763.3 pmol/L. These results indicated a mild elevation of epinephrine, with the concentration remaining below twice the upper limit of the normal reference range. Plasma free metanephrines and urinary fractionated metanephrines were both within normal reference ranges. Contrast-enhanced computed tomography (CT) of the lower abdomen and pelvis (**Figure 1**) demonstrated a well-circumscribed, slightly hypoattenuating nodule measuring 2.6 × 2.1 cm located inferior to the left adrenal gland and medial to the midpole of the left kidney. On post-contrast images, the lesion exhibited prominent nodular and peripheral ring enhancement (**Figures 1A1, 1A2**). The right adrenal gland displayed diffuse thickening with multiple isoattenuating nodules, the largest measuring 1.8 cm in diameter (**Figures 1B1, 1B2**). Bilateral adrenal nodules were thus identified. Given the clinical context, pheochromocytoma or recurrent chromaffin neoplasm was considered the leading differential diagnosis, although adrenal adenoma and other adrenal lesions could not be definitively excluded. Due to financial constraints, the patient declined resection of the right adrenal mass and consented only to excision of the left adrenal lesion. Additionally, the patient declined all supplementary examinations, including metaiodobenzylguanidine (MIBG) scintigraphy, PET-CT, somatostatin receptor scintigraphy, and genetic testing. Partial adrenalectomy was chosen as the preferred surgical approach due to its minimally invasive nature. This technique allows complete tumor enucleation while preserving residual adrenocortical function and is associated with favorable postoperative recovery. Preoperative α-adrenergic blockade was achieved with oral phenoxybenzamine to promote vasodilation. Subsequently, partial left adrenalectomy was performed for the left adrenal tumor. Intraoperative exploration revealed adhesions involving the descending colon, peritoneum, and spleen. Two solid nodules were identified in the left periadrenal region adjacent to the renal hilum; the larger lesion measured 2 cm × 2 cm and exhibited dense adhesions to the pancreatic parenchyma. No significant intraoperative hemodynamic fluctuations were observed. The specimen was submitted for pathological examination.

Gross Examination: Two irregular fragments of grayish-red tissue with attached fatty soft tissue were received. The larger fragment measured 3.2 cm × 2.5 cm × 1.5 cm and had been incised intraoperatively. Sectioning revealed a cystic space approximately 2 cm in diameter with evacuated luminal contents; the cyst wall ranged from 0.2 to 0.4 cm in thickness and exhibited smooth internal and external surfaces. The smaller fragment measured 2.0 cm × 1.8 cm × 1.5 cm and was covered by an intact capsule. Its cut surface showed solid, grayish-red to grayish-yellow parenchyma of moderate consistency.

**Figure 2:**
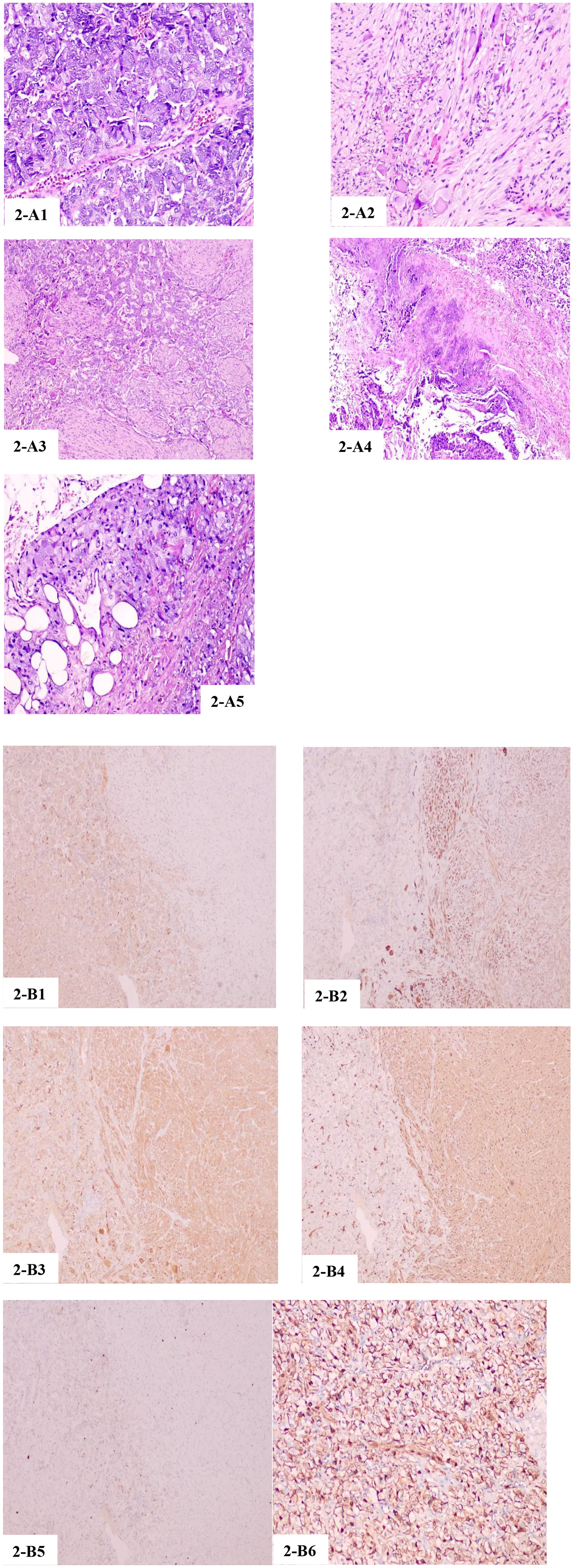
Histomorphological characteristics and immunohistochemical profiles of CP–GN. Immunohistochemical staining was performed via the EnVision detection system (Zhongshan Jinqiao Biotechnology Co., Ltd., Beijing, China). **(A1)** Pheochromocytoma component (original magnification ×100): Tumor cells grow in characteristic nested zellballen architecture. Lesional cells exhibit pleomorphic polygonal or spindled morphology with abundant finely granular basophilic to amphophilic cytoplasm; focal cells possess clear cytoplasm, and mitoses are infrequent. The stroma is highly vascularized. **(A2)** GN component (×100): Uniform spindled Schwann cells infiltrate collagenous stroma arranged in fascicles with wavy nuclei; scattered ganglion cells of variable size are interspersed throughout the stroma. **(A3)** Interface of the two neoplastic components (×40): Pheochromocytomatous and ganglioneuromatous tissues interdigitate without a distinct demarcating border. (**A4)** Tumor necrotic foci (×100). (**A5)** Invasion of peritumoral adipose tissue by the pheochromocytoma component (×100). (**B1)** CgA staining at the tumor interface (×40): Diffuse positive immunoreactivity is seen within the pheochromocytoma compartment, whereas the ganglioneuromatous compartment shows negative staining. (**B2)** NF staining at the tumor interface (×40): The ganglioneuromatous component demonstrates widespread positive staining, while the pheochromocytoma component yields negative results. (**B3)** NSE staining at the tumor interface (×40): Uniform positive immunoreactivity is present across the ganglioneuromatous areas, and only focal positive staining is detected within pheochromocytoma regions. (**B4)** S100 protein staining at the tumor interface (×40): Diffuse positivity is noted throughout ganglioneuromatous stroma; in pheochromocytoma areas, staining is limited to the surrounding sustentacular cell network. (**B5)** Ki-67 proliferation index at the tumor interface(average-field) (×40): Less than 3% of cells within the pheochromocytoma compartment display positive nuclear Ki-67 staining, and 3% of ganglioneuromatous cells show Ki-67 positivity. (**B6)** S100 protein staining highlighting the sustentacular cell network within the pheochromocytoma component (×100).*CP* composite pheochromocytoma *CgA* Chromogranin A *GN* ganglioneuroma *NF* Neurofilament *NSE* Neuron-specific enolase.

On H&E staining, two morphologically distinct neoplastic components, arranged as separate nodules, were delineated by fibrous septa and exhibited focal intermingling without sharp demarcation (**Figure 2A3**). One compartment comprised tumor cells organized in a characteristic nested “zellballen” architecture. These cells displayed pleomorphic polygonal and spindled morphology, with abundant finely granular basophilic to amphophilic cytoplasm; occasional cells harbored clear cytoplasm, and mitotic figures were scarce. This region was highly vascularized (**Figure 2A1**). Additional histologic alterations included cystic degeneration, multifocal neoplastic necrosis (**Figure 2A4**), and infiltration into the surrounding peritumoral adipose tissue (definite invasion) (**Figure 2A5**). The second compartment consisted of relatively monotonous spindled Schwann cells embedded within a collagen-rich stroma, growing in fascicles and cords, and characterized by elongated, wavy nuclei. Scattered ganglion cells of variable size were dispersed throughout this stromal background (**Figure 2A2**).

Immunohistochemical staining(EnVision method, Beijing Zhongshan Jinqiao Biotechnology Co., Ltd., **Figure 2B**): In the pheochromocytoma component, chromogranin A (CgA) exhibited diffuse positive staining, while synaptophysin (Syn) demonstrated diffuse immunoreactivity. Neuron-specific enolase (NSE) showed focal positivity. S100 protein (S100) was detected in the surrounding sustentacular cells, and SRY-box transcription factor 10 (SOX10) was likewise positive within this cell population. Immunostaining for melanoma antigen recognized by T cells 1 (MART-1/MelanA) was negative, as were neurofilament light and heavy chains (NF-L+H). Smooth muscle actin (SMA) revealed no positive staining, and inhibin alpha subunit (inhibin-α) was negative. Pan-cytokeratin (pCK) was entirely non-reactive. The Ki-67 proliferation index was positive in fewer than 3% of tumor cells (average field).

In the GN component, chromogranin A (CgA) showed focal, weak positivity, whereas melanoma antigen recognized by T cells 1 (MART-1/MelanA) exhibited diffuse immunoreactivity. Neurofilament light and heavy chains (NF-L+H) were diffusely positive, and neuron-specific enolase (NSE) displayed widespread staining. S100 protein (S100) demonstrated diffuse positivity throughout the stroma. Smooth muscle actin (SMA) was focally positive, and SRY-box transcription factor 10 (SOX10) showed diffuse immunoreactivity, while synaptophysin (Syn) yielded positive staining. Inhibin alpha subunit (inhibin-α) remained negative, and pan-cytokeratin (pCK) was entirely non-reactive. The Ki-67 proliferation index was positive in 3% of tumor cells (average field). Final histopathological diagnosis confirmed CP consisting of approximately 80% pheochromocytoma component and 20% GN component. Surgical margin status could not be definitively assessed due to intraoperative specimen fragmentation. At 11-month telephone follow-up, the patient remained asymptomatic; however, scheduled imaging and biochemical evaluations were not completed.

## Discussion

Per the 2022 fifth edition of the World Health Organization (WHO) classification of endocrine and neuroendocrine tumors, pheochromocytomas and paragangliomas (PPGLs) are classified as non-epithelial neuroendocrine neoplasms (NENs) with heterogeneous metastatic potential. Notably, the WHO no longer classifies PPGLs into benign and malignant subtypes, as all PPGLs carry variable risks of local recurrence or distant metastasis. The biological behaviors of PPGLs are analogous to those of epithelial NENs; accordingly, lesions presenting with distant metastases are defined as metastatic PPGLs (8). Clinically, composite PPGLs pose a considerable diagnostic challenge in distinguishing them from conventional pure PPGLs (9), and more than 75% of these composite tumors are hormonally functional (10, 11). While isolated ganglioneuromas are typically endocrinologically silent and clinically asymptomatic, multiple published cases have described functional ganglioneuromas capable of secreting a broad spectrum of bioactive substances, including catecholamines and their metabolites, as well as, rarely, adrenocorticotropic hormone, cortisol, androgens, and vasoactive intestinal peptide (12).Adrenal medullary composite tumors exhibit highly variable clinical phenotypes, and a substantial proportion of patients present without hypertension or classic catecholamine-related manifestations (13). Consistent with this clinical pattern, our patient lacked hallmark pheochromocytoma-associated symptoms; nearly all serum hormone markers remained within the reference ranges, with only a mild isolated elevation of epinephrine. The mechanisms underlying the absence of endocrinopathy and typical pheochromocytoma symptoms in a subset of composite adrenal tumors have yet to be fully elucidated.

Differential diagnosis for CP admixed with ganglioneuroma primarily includes pure pheochromocytoma, isolated ganglioneuroma, and pediatric neuroblastic tumors such as NB/GNB (7, 14, 15). Pure pheochromocytoma is devoid of mature ganglion cells, whereas ganglioneuroma lacks chromaffin cell nests and rarely leads to elevated metanephrine levels (14). The primitive neuroblastic precursors characteristic of neuroblastoma are absent in this well-differentiated composite lesion. Immunohistochemistry facilitates this distinction: chromogranin A and synaptophysin highlight the chromaffin component, neurofilament identifies ganglion cells, and S100 delineates the Schwannian stroma (7). In a cohort study by Zawadzka et al., patients with CP were significantly older than those with conventional pure pheochromocytoma, with median ages of 67 and 56 years, respectively (P = 0.015) (15). Furthermore, elevated Ki-67 proliferation indices (3%–5%) represented an independent high-risk factor for composite lesions (odds ratio, 28.68; 95% confidence interval, 1.49–552.79; P = 0.026) (15). However, tumor size, cumulative recurrence and metastatic rates, and overall mortality were comparable between the composite and typical pheochromocytoma groups.

The pathogenesis of composite PPGLs has not been fully elucidated. Recent studies have identified that driver gene mutations associated with hereditary PPGLs—including NF1, RET, VHL, and MAX—are also present in composite PPGL lesions, suggesting shared pathogenic mechanisms between conventional PPGLs and their composite subtypes (16). Furthermore, composite PPGLs are known to be associated with several hereditary tumor syndromes, such as multiple endocrine neoplasia type 2 (MEN2), neurofibromatosis type 1 (NF1), and related genetic disorders (17). In the present case, genetic testing was not performed. Although the patient denied any relevant familial history of hereditary neoplasms or a personal diagnosis of genetic tumor syndromes, the potential contribution of underlying gene mutations or inherited syndromes cannot be completely excluded.

Currently, for PPGLs without initial distant metastasis, radical resection remains the main treatment method, but the prognosis assessment and metastasis risk judgment of postoperative PPGLs still face significant challenges. Whether there are differences in treatment strategies and prognostic significance between CP and simple pheochromocytoma remains unclear.

From a prognostic standpoint, a core clinical challenge in managing PPGLs lies in accurately stratifying individual risks of subsequent distant metastasis. In this context, metastatic PPGLs must be clearly distinguished from multifocal synchronous primary PPGLs. Criteria defining metastatic PPGLs rely on anatomical distribution relative to the autonomic nervous system. Per the 2022 WHO endocrine tumor classification, only bone and non-regional lymph nodes are recognized as definitive metastatic sites; the guideline further highlights diagnostic pitfalls that may blur the distinction between metastatic lesions and multifocal primary paragangliomas at other anatomic locations (14).

The 2022 WHO guidelines recommend S100 and SOX10 immunohistochemistry as an auxiliary diagnostic tool for this differential workup. Multifocal primary PPGLs feature an intact, interconnected network of sustentacular cells throughout the lesion, whereas sustentacular cell populations are markedly diminished in biologically aggressive paragangliomas (18). A robust sustentacular cell network serves as a key morphologic marker favoring multifocal primary disease over metastasis, as metastatic deposits typically lack well-organized sustentacular cell architecture. In our case, S100 staining delineated a complete intratumoral sustentacular cell network, consistent with a primary adrenal neoplasm (**Figure 2B6**).

In 2019, Pierre et al. from France proposed the CP/Paraganglioma Prognostic Score (COPPS), an integrated risk stratification tool for PPGLs. This scoring system incorporates five predictive variables: one clinical parameter (tumor diameter > 7 cm), two high-risk histological features (tumor necrosis and vascular invasion), loss of S100 expression (indicative of sustentacular cell depletion), and loss of SDHB immunoreactivity (suggesting underlying pathogenic variants in succinate dehydrogenase subunit genes (SDHx)). Tumor necrosis is assigned a weighted score of 5 points, whereas the remaining four criteria each contribute 1–2 points, yielding a maximum total score of 10.A COPPS score ≥ 3 correlates with shortened progression-free survival (PFS) and demonstrates excellent predictive performance for distant metastasis, with a reported sensitivity of 100% and specificity of 95%. Qin et al. (19) recently conducted a comparative validation of five risk-stratification tools for predicting metastatic potential in PPGLs, including the Pheochromocytoma of the Adrenal Gland Scaled Score (PASS), Grading of Adrenal Pheochromocytoma and Paraganglioma (GAPP), COPPS, the Age, Size, Extra-adrenal Location, Secretory type (ASES) score, and the Size, Genetic status, Age, PASS (SGAP) model. Their analysis demonstrated that the COPPS system achieved the highest C-index for predicting metastatic propensity; however, its generalizability remains to be confirmed through independent external validation in larger patient cohorts (22).

Multifocal tumor necrosis was observed in the present case, despite a maximum tumor diameter of less than 7 cm and the absence of vascular invasion. Immunohistochemical staining for S100 demonstrated intact sustentacular cell architecture without complete loss. As SDHB immunohistochemical staining was not available at our institution, the COPPS score was calculated to be 5 points without incorporating the SDHB criterion. Even with positive SDHB immunohistochemical staining, the COPPS total score remains above 3 points—indicating a potentially adverse prognosis characterized by shortened progression-free survival and elevated risk of metastasis. While potentially useful, COPPS has limited validation and has not been specifically established in CP, more research is still needed in the future to validate its rationality.

Furthermore, a prior cohort study (20) demonstrated that a Ki-67 proliferation index ≥3% is significantly associated with increased risks of disease progression, local recurrence, and distant metastasis in patients with PPGLs. In this case, the tumor exhibited a Ki-67 index of 3%, which may indicate high-risk histopathological and prognostic features associated with an elevated risk of adverse oncologic outcomes, warranting long-term surveillance with defined imaging and biochemical intervals.

Metastatic progression in CP is exceedingly rare, with only isolated case reports documented in the literature (4); in contrast, NB is intrinsically malignant and consistently exhibits aggressive biological behavior (21). Notably, this patient had undergone surgical resection of a conventional ipsilateral adrenal pheochromocytoma 13 years earlier. The left adrenal lesion in this case may represent either late ipsilateral recurrence of the initial chromaffin neoplasm or a metachronous *de novo* primary CP arising independently within the same gland. Concurrently, imaging findings raised strong suspicion for a synchronous contralateral primary pheochromocytoma. Collectively, these features indicate that the patient faces lifelong risks of ipsilateral local recurrence, *de novo* ipsilateral adrenal neoplasms, and metachronous contralateral primary adrenal tumors.

Several limitations of this study warrant acknowledgment. First, the absence of archival histopathologic slides from the index surgery precluded direct microscopic comparison with the recurrent lesion, thereby limiting definitive confirmation of true tumor recurrence. Second, hereditary pheochromocytoma–paraganglioma syndrome could not be ruled out, as comprehensive germline genetic testing was not performed—primarily due to financial barriers reported by the patient. Third, the histopathologic characterization of the contralateral adrenal mass remains undetermined, as the patient declined surgical resection of the right adrenal lesion for socioeconomic reasons. Finally, the study was limited by a relatively short follow-up duration and incomplete surveillance: the patient did not undergo scheduled imaging or biochemical evaluations during the 11-month monitoring period.

## Conclusion

Pheochromocytomas containing ganglioneuromatous components represent a rare subtype of PPGLs. Their preoperative clinical presentations and imaging features closely resemble those of conventional, histologically pure pheochromocytomas—posing significant challenges for accurate preoperative differential diagnosis. Moreover, published case series remain limited, and the biological behavior and underlying genetic landscape of this entity remain incompletely characterized. Although most patients exhibit an indolent clinical course with favorable long-term outcomes, these tumors retain inherent metastatic potential. To date, no clinically validated biomarker—whether clinical or histopathological—has been established to reliably predict metastatic progression. In contrast, integrated risk stratification—incorporating intratumoral histologic composition, germline and somatic genomic profiling. In addition, exploratory auxiliary risk stratification tools such as COPPS can be consulted for reference for estimating metastatic risk and predicting long-term survival. This multimodal assessment framework informs the development of individualized therapeutic strategies and underpins the design of evidence-based, standardized long-term surveillance protocols.

## Data Availability

The original contributions presented in the study are included in the article. Further inquiries can be directed to the corresponding author.
